# A resected case of two branch duct-type intraductal papillary mucinous neoplasms showing different clinical courses after a two-year follow-up

**DOI:** 10.1007/s12328-017-0728-1

**Published:** 2017-03-03

**Authors:** Hideki Shibata, Nobuyuki Ohike, Tomoko Norose, Tomohide Isobe, Reika Suzuki, Hideyuki Imai, Akira Shiokawa, Masafumi Takimoto, Akihiro Tabuchi, Yuichi Takano, Eiichi Yamamura, Masatsugu Nagahama, Nobuyuki Takeyama, Kazuaki Yokomizo, Hiroki Mizukami, Jun-ichi Tanaka, Takeshi Aoki, Masahiko Murakami

**Affiliations:** 1 Department of Pathology, Showa University Fujigaoka Hospital, 1-30 Fujigaoka, Aoba-ku, Yokohama, 227-8501 Kanagawa Japan; 2 Department of Pathology, Showa University Hospital, Shinagawa, Tokyo Japan; 3 Division of Gastroenterology, Department of Internal Medicine, Showa University Fujigaoka Hospital, Yokohama, Kanagawa Japan; 4 Department of Radiology, Showa University Fujigaoka Hospital, Yokohama, Kanagawa Japan; 5 Department of Gastroenterological and General Surgery, Showa University Fujigaoka Hospital, Yokohama, Kanagawa Japan; 6 Department of Gastroenterological and General Surgery, Showa University Hospital, Shinagawa, Tokyo Japan

**Keywords:** Brunch duct-type IPMN, Malignant mural nodule, Simple mucinous cyst, KRAS mutation

## Abstract

The patient was a 60-year-old man without any particular complaints, but he underwent abdominal computed tomography (CT) and magnetic resonance cholangiopancreatography (MRCP) due to a fatty liver, which revealed two similar cystic lesions regarded as branch duct-type intraductal papillary mucinous neoplasm (BD-IPMN) in the pancreatic body [BD-IPMN (*b*), 16 mm in size] and tail [BD-IPMN (*t*), 13 mm in size] without a “high-risk stigmata” or “worrisome features”. He subsequently received follow-up by MRCP every 6 months. Two years later, MRCP showed prominent dilation of the main pancreatic duct (MPD) and mural nodule formation within the dilated MPD adjacent to the BD-IPMN (*b*). Distal pancreatectomy specimens revealed that the BD-IPMN (*b*) was lined by low-papillary gastric mucinous epithelium with low-to-intermediate-grade dysplasia and involved the MPD, forming a malignant mural nodule showing pancreatobiliary-type IPMN. In contrast, the BD-IPMN (*t*) was lined by flat, monolayer columnar gastric mucinous epithelium without atypia, which suggested the possibility of a “simple mucinous cyst”. A genetic analysis showed KRAS mutation only in BD-IPMN (*b*). Differences in the histological and genetic findings between two similar BD-IPMNs in the present case may suggest what kinds of examinations should be performed in patients with BD-IPMNs without any worrisome features.

## Introduction

Intraductal papillary mucinous neoplasm (IPMN) has variable malignant potential, ranging from premalignant intraductal lesions to malignant neoplasms with invasive carcinoma. IPMNs are classified according to their ductal involvement into three types: main duct (MD)-IPMN, branch duct (BD)-IPMN, and mixed-type-IPMN [1, 2]. BD-IPMN is the most common and often diagnosed incidentally and has the lowest risk of malignancy; a natural history study estimates the risk for BD-IPMN progressing to high-grade dysplasia to be less than 3% [3]. As such, many BD-IPMNs can be managed conservatively, and a precise diagnosis of malignancy is strongly required to avoid unnecessary surgeries.

The international consensus guidelines for the management of IPMN, which were first formulated in 2006 (‘Sendai consensus guidelines’) [1] and subsequently revised in 2012 (‘Fukuoka consensus guidelines’) [4], stratified BD-IPMNs into three categories: high risk with “high-risk stigmata”, worrisome risk with “worrisome features”, and low risk [4]. The Fukuoka guidelines suggested that low-risk BD-IPMNs without any “worrisome features” [cyst of ≥3 cm, thickened enhanced cyst walls, main pancreatic duct (MPD) diameter size of 5–9 mm, non-enhanced mural nodules, and abrupt change in the MPD caliber with distal pancreatic atrophy] should be observed without immediate resection. Based on the cyst size and features of presentation, computed tomography (CT), magnetic resonance imaging (MRI) and endoscopic ultrasonography (EUS) are the chosen methods of surveillance. The Fukuoka guidelines propose sequential imaging surveillance alternating MRI and EUS as often as every 3–6 months in the initial phase of surveillance for BD-IPMN patients with cysts of >2 cm, while longer interval (12–36 months) surveillance is recommended for those with cysts of <2 cm [4]. However, the frequency of surveillance for BD-IPMN patients is currently controversial [5].

We herein report a surgically resected case of two small, low-risk BD-IPMNs, one of which involved the MPD and formed a malignant mural nodule and the other which was almost stable except for cyst enlargement during 2 years of follow-up. The pathological and biological differences between the two BD-IPMNs are discussed.

## Case report

**Fig. 1:**
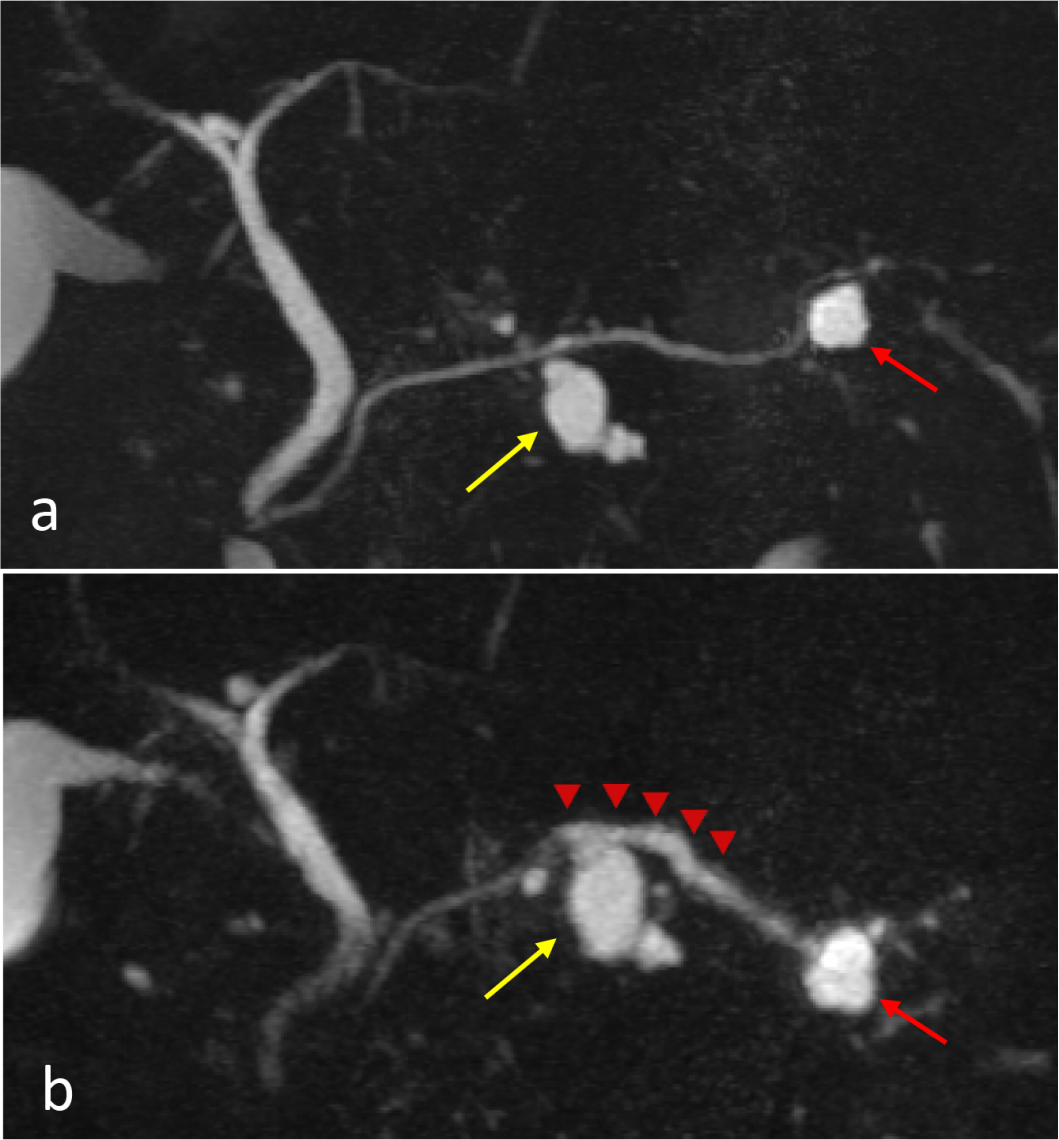
*MRCP* showed BD-IPMNs in the pancreatic body (indicated by a *yellow arrow*) and tail (indicated by a *red arrow*) at the initial visit (**a**); prominent dilation of the MPD (indicated by *red arrowheads*) and enlargement of the cystic lesions were observed 2 years later (**b**)

The patient was a 60-year-old man without any particular complaints. There is no remarkable information on his past and life history. He underwent abdominal CT and magnetic resonance cholangiopancreatography (MRCP) due to a fatty liver pointed out during a health examination, and two small cystic lesions adjacent to the MPD of the pancreas measuring 16 mm in the body and 13 mm in the tail were detected (Fig. 1a). Although the communication between cysts and MPD was obscure, they were regarded as multifocal BD-IPMNs without high-risk stigmata or worrisome features, and he subsequently received careful follow-up by MRCP every 6 months.

**Fig. 2:**
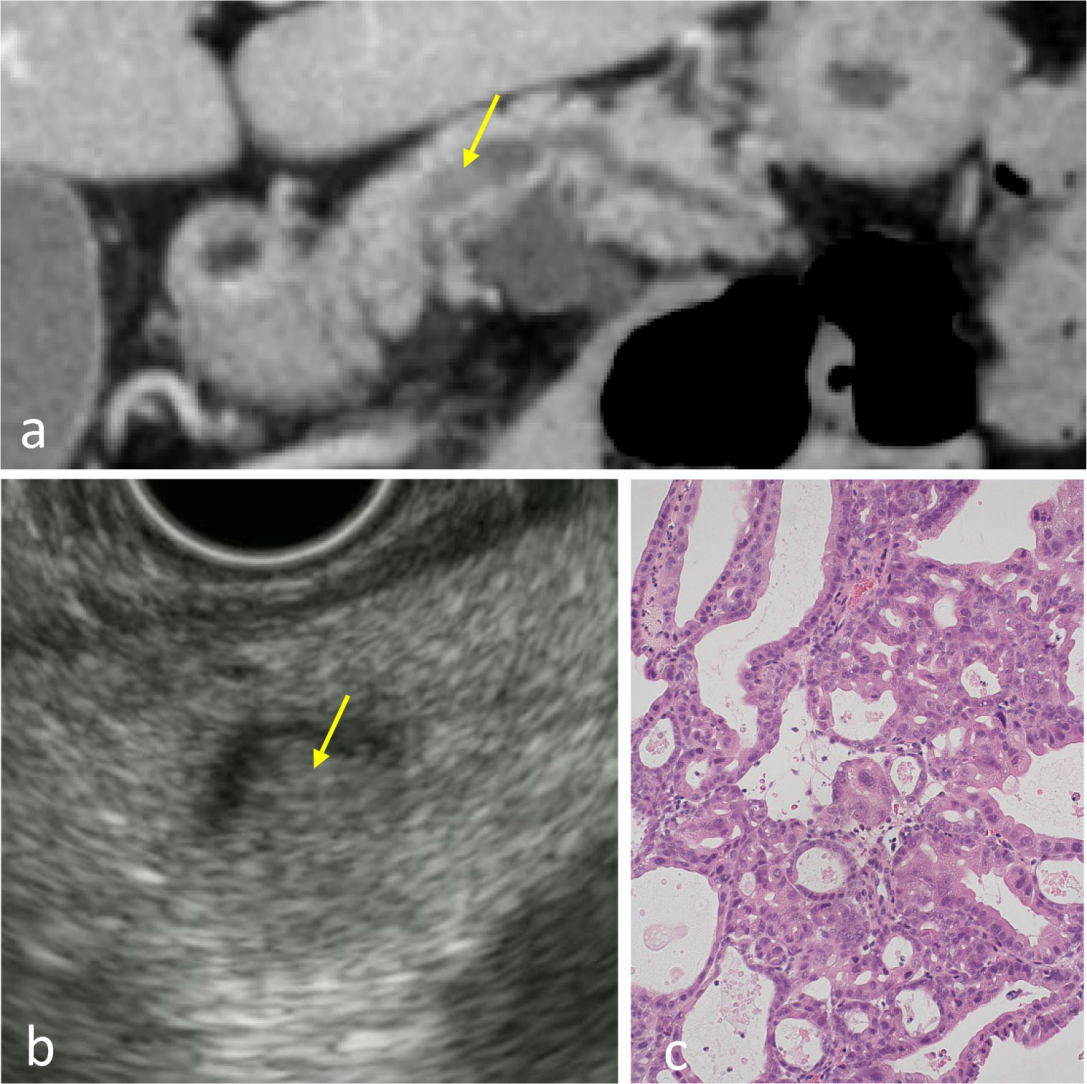
Enhanced CT (**a**) showed a mural nodule (indicated by a *yellow arrow*) within the dilated MPD adjacent to the BD-IPMN in the pancreatic body, corresponding to a *low*-*echoic tumor* (mural nodule; indicated by a *yellow arrow*) within the MPD by EUS (**b**). The endoscopic biopsy of the mural nodule revealed adenocarcinoma (**c**)

After 2 years, MRCP showed an enlargement (16–21 mm) of the BD-IPMN of the body [BD-IPMN (*b*)] and a prominent dilation of the adjacent MPD measuring 5 mm (Fig. 1b). Abdominal enhanced CT revealed a mural nodule within the dilated MPD, corresponding to a low-echoic tumor (8 mm) by EUS (Fig. 2a, b). The prominent dilation of the MPD and mural nodule were not visible in previous MRCP images. An endoscopic transpapillary forceps biopsy (FB-39Q-1, 1.95 mm, OLYMPAS, Japan) of the mural nodule revealed adenocarcinoma (Fig. 2c). The BD-IPMN of the tail [BD-IPMN (*t*)] showed only enlargement (13–19 mm). Blood tumor markers (CEA, CA19–9, and DUPAN-2) were within normal ranges. The patient underwent distal pancreatectomy for IPMNs with a malignant mural nodule.

**Fig. 3:**
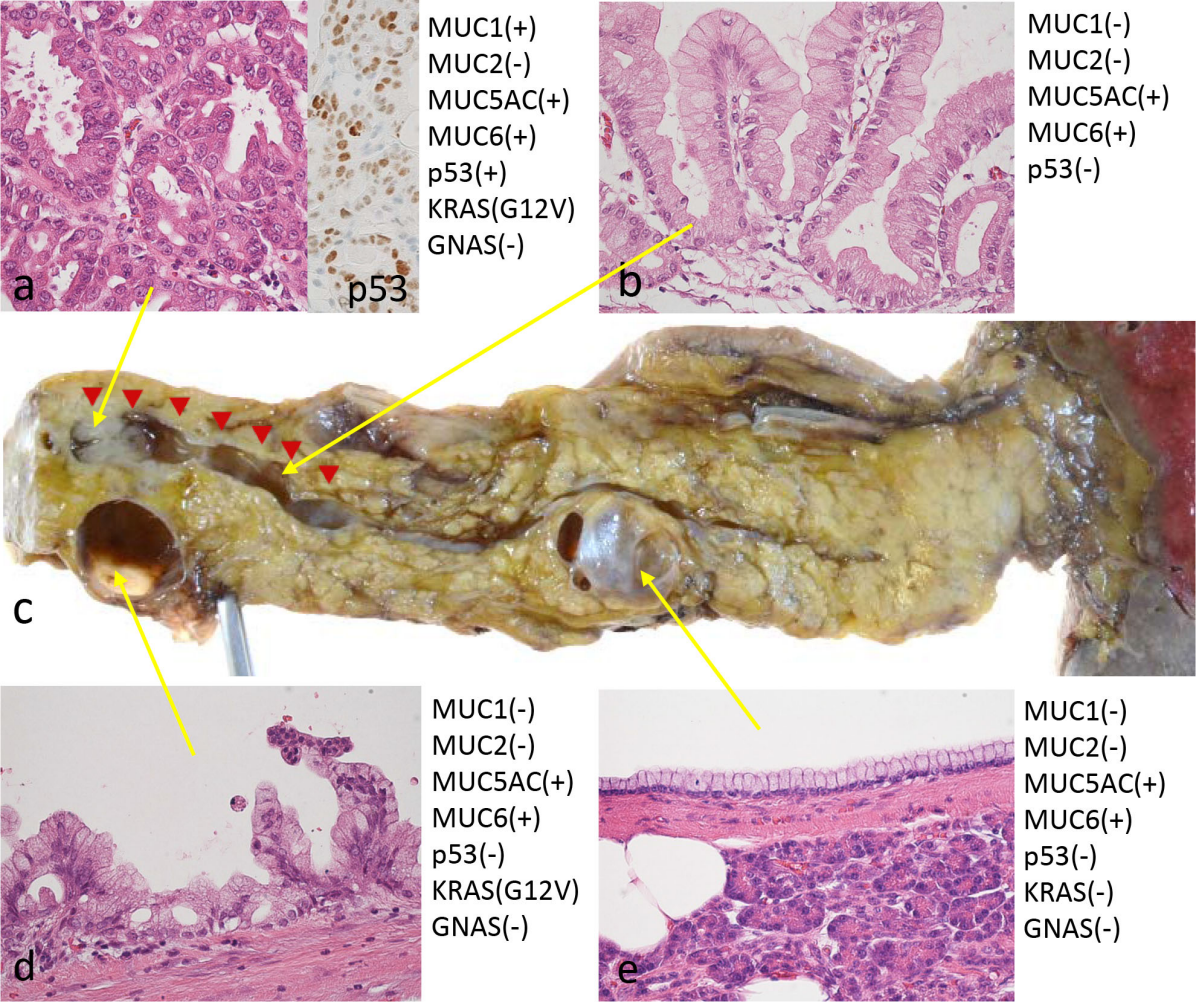
The resected specimen (**c**) revealed that the mural nodule in the MPD consisted of PB-type IPMN with high-grade dysplasia (adenocarcinoma) (**a**) with a diffuse positivity of p53 immunostaining (a* insert*) and KRAS mutation (G12V). The BD-IPMN of the body was lined by gastric mucinous epithelium showing *low* papillary configuration with mild epithelial stratification with the same KRAS mutation (**d**), and the proliferation of similar gastric IPMN components sequentially involved the *bottom* of the mural nodule and the wall of the surrounding dilated MPD (indicated by *red arrowheads*) (**b**). The BD-IPMN of the tail was lined by *flat*, monolayer gastric mucinous epithelium lacking cellular atypia and KRAS mutation (**e**)

Macroscopically, both BD-IPMNs showed flat cystic lesions without visible papillary lesions, but the BD-IPMN (*b*) contained more viscous mucin. The dilated MPD (MD-IPMN) adjacent to the BD-IPMN (*b*) included the mural nodule (measuring 12 × 8 mm) filling the lumen and was associated with thickening of the duct wall (Fig. 3c).

Microscopically, the BD-IPMN (*b*) was lined by tall columnar epithelium with oval nuclei and clear mucinous cytoplasm showing flat to papillary configurations with mild epithelial stratification and low to intermediate cellular dysplasia (Fig. 3d). The immunohistochemistry (IHC) showed a gastric lineage (MUC5AC+, MUC6+, MUC1− and MUC2−) and was CA19-9-positive, CEA-negative, p53-negative, and had a very low Ki-67 index (<2%). The proliferation of similar gastric IPMN components sequentially involved the bottom of the mural nodule and the wall of the surrounding dilated MPD (Fig. 3b; the affected region was indicated by arrow heads in Figs. 1b and 3c). The mural nodule consisted mainly of cuboidal epithelial cells with oval nuclei and eosinophilic cytoplasm forming complex arborizing papillae, suggestive of high-grade dysplasia (carcinoma; Fig. 3a). The IHC showed a pancreatobiliary lineage (MUC5AC+, MUC6+, MUC1+ and MUC2−) and was CA19-9-positive, CEA focally positive, p53-positive (overexpression of p53 protein; Fig. 3a), and had a low Ki-67 index (5%). No invasive carcinoma components were found. All of these lesions shared the same genetic characteristics: KRAS mutation (G12V)-positive and GNAS mutation-negative.

In contrast, the BD-IPMN (*t*) was lined by flat, monolayer columnar epithelium with basally located nuclei and mucinous cytoplasm lacking cellular atypia (Fig. 3e). IHC showed a gastric lineage (MUC5AC+, MUC6+, MUC1− and MUC2−) that was CA19-9-positive, CEA-negative, p53-negative, and had a very low Ki-67 index (<2%). A genetic analysis showed no mutations of KRAS or GNAS.

## Discussion

The current case presents two issues to be discussed. First, we must address the relationship between the pre-existing BD-IPMN (*b*) and MD-IPMN with mural nodule complicated after 2 years—specifically, whether MD-IPMN was merely concomitant and located nearby or had continuously developed from the BD-IPMN (*b*). Indeed, the communication between the BD-IPMN (*b*) and MPD was not clear on the initial images, but the sequential progression from the BD-IPMN (*b*) to MD-IPMN seems to be more likely than concomitant development for the following reasons: (1) resected specimens revealed totally sequential spread between the BD-IPMN (*b*) and MD-IPMN; (2) the IPMN components of the thickened wall of the MPD and the bottom of the mural nodule showed a similar cell lineage (gastric), cellular dysplasia (mild to intermediate), and genetic characteristics [same KRAS mutation (G12V)] to the BD-IPMN (*b*); and (3) PB-type IPMN with high-grade dysplasia comprised the mural nodule, which was probably transformed from lower-grade gastric IPMN [6], shared the same genetic characteristics [KRAS mutation (G12V)]. As for the growth speed of the tumor, the mural nodule may have been formed within 6 months because visible changes were not detected in the previous MRCP; although invisible, small lesions may have been more previously latent. Mural nodule formation in such a short term may be one of the features when lower-grade, gastric-type BD-IPMN involves focal high-grade transformation of PB-type IPMN, but further surveillance studies of similar cases will be required.

The second point of discussion is the significance of the histological and biological differences between the BD-IPMN (*b*) and BD-IPMN (*t*) in the same patient’s pancreas, which may reveal noteworthy factors for the effective surveillance of BD-IPMNs without worrisome features. The BD-IPMN (*b*) and BD-IPMN (*t*) in the present patient were almost identical cysts on the initial images. However, while the BD-IPMN (*b*) progressed to a higher-grade MD-IPMN, requiring resection, the BD-IPMN (*t*) showed only enlargement during the follow-up period. The resected specimens provided few but considerable findings, as follows: although both BD-IPMNs showed the same gastric phenotype, the BD-IPMN (*b*) was lined by mild to intermediate dysplastic epithelium showing low papillae and mild epithelial stratification, while the BD-IPMN (*t*) was lined by a very low-grade dysplastic epithelium without overt papillary configuration. BD-IPMN (*t*) might be classified as a “simple mucinous cyst”, which has been recently proposed as a mimicker of BD-IPMN or mucinous cystic neoplasm and rarely develops malignancy [7, 8]. In addition, the BD-IPMN (*b*) showed a KRAS mutation (G12V), but the BD-IPMN (*t*) did not. The KRAS mutation is typically detected in low-grade pancreatic ductal lesions, and its influence on preoperative patient management has yet to be determined. However, these findings indicate that not only the cyst size and morphology on imaging, cytology, and serum tumor markers, but also other criteria such as biochemical and molecular factors may be useful in selecting BD-IPMNs for resection.
